# Potential Arrhythmogenic Role of TRPC Channels and Store-Operated Calcium Entry Mechanism in Mouse Ventricular Myocytes

**DOI:** 10.3389/fphys.2018.01785

**Published:** 2018-12-13

**Authors:** Hairuo Wen, Zhenghang Zhao, Nadezhda Fefelova, Lai-Hua Xie

**Affiliations:** ^1^Department of Cell Biology and Molecular Medicine, Rutgers New Jersey Medical School, Newark, NJ, United States; ^2^Key Laboratory of Beijing for Nonclinical Safety Evaluation Research of Drugs, National Center for Safety Evaluation of Drugs, National Institutes for Food and Drug Control, Beijing, China; ^3^Department of Pharmacology, School of Basic Medical Sciences, Xi’an Jiaotong University, Xi’an, China

**Keywords:** calcium, arrhythmogenesis, TRPCs, store-operated calcium entry, hyperforin

## Abstract

**Background and Purpose:** Store-operated calcium entry (SOCE) is an important physiological phenomenon that extensively mediates intracellular calcium ion (Ca^2+^) load. It has been previously found in myocytes isolated from neonatal or diseased hearts. We aimed to determine its existence, molecular nature in undiseased hearts and its potential arrhythmogenic implications under hyperactive conditions.

**Experimental Approach:** Ventricular myocytes isolated from adult FVB mice were studied by using Ca^2+^ imaging and whole-cell perforated patch-clamp recording. In addition, lead II ECGs were recorded in isolated Langendorff-perfused mice hearts. Functional TRPC channel antibodies and inhibitors, and TRPC6 activator hyperforin were used.

**Key Results:** In this study, we demonstrate the existence and contribution of SOCE in normal adult mouse cardiac myocytes. For an apparent SOCE activation, complete depletion of sarcoplasmic reticulum (SR) Ca^2+^ by employing both caffeine (10 mM) and thapsigargin (1 μM) or cyclopiazonic acid (10 μM) was required. Consistent with the notion that SOCE may be mediated by heteromultimeric TRPC channels, SOCEs observed from those myocytes were significantly reduced by the pretreatment with anti-TRPC1, 3, and 6 antibodies as well as by gadolinium, a non-selective TRPC channel blocker. In addition, we showed that SOCE may regulate spontaneous SR Ca^2+^ release, Ca^2+^ waves, and triggered activities which may manifest cardiac arrhythmias. Since the spontaneous depolarization in membrane potential preceded the elevation of intracellular Ca^2+^, an inward membrane current presumably via TRPC channels was considered as the predominant cause of cellular arrhythmias. The selective TRPC6 activator hyperforin (0.1–10 μM) significantly facilitated the SOCE, SOCE-mediated inward current, and calcium load in the ventricular myocytes. ECG recording further demonstrated the proarrhythmic effects of hyperforin in *ex vivo* mouse hearts.

**Conclusion and Implications:** We suggest that SOCE, which is at least partially mediated by TRPC channels, exists in adult mouse ventricular myocytes. TRPC channels and SOCE mechanism may be involved in cardiac arrhythmogenesis via promotion of spontaneous Ca^2+^ waves and triggered activities under hyperactivated conditions.

## Introduction

Intracellular/cytosolic calcium ion (Ca^2+^) is crucial in regulating various fundamental cellular processes (Berridge, 1993; Carafoli, 2002), while dysfunction of Ca^2+^ handling leads to pathological consequences such as cardiac arrhythmias and hypertrophy (Wit and Janse, 1992). Elevation in Ca^2+^ concentration can be mediated by a process referred as SOCE in many cell types, i.e., an extracellular Ca^2+^ influx through plasma membrane channels triggered by an excessive release/depletion of Ca^2+^ from ER or SR stores (Putney, 1986; Selvaraj et al., 2010). SOCE has been identified as a major process for increasing Ca^2+^ load after the ER/SR Ca^2+^ depletion in eukaryotic cells (Parekh and Putney, 2005). Although SOCE has also been found in myocytes isolated from neonatal and diseased hearts (Uemura et al., 2002; Huang et al., 2006; Sabourin et al., 2016; Ross et al., 2017), its existence, molecular nature in undiseased hearts and its potential arrhythmogenic implications under hyperactive conditions remain elusive.

Transient receptor potential canonical (TRPC) channels are a family of non-selective cytoplasmic channels distributed throughout the cardiac myocytes, and six subtypes (TRPC1 and 3-7) have been identified in human ventricles (Venkatachalam and Montell, 2007). TRPC channels contribute to both Ca^2+^ influx and inward membrane currents, which in turn could mediate cellular signaling and Ca^2+^ homeostasis (Liao et al., 2009). The participations of TRPC channels in calcium signaling in the form of SOCE were primarily revealed in a heterogeneous expression system, in which ER/SR depletion-activated Ca^2+^ entry was significantly enhanced by the over-expression of TRPC subtypes, and reduced by pharmacological and genetic knockdown approaches (Liao et al., 2009). Recent studies have further proposed that the TRPC channels constitute SOCE by forming complex together with calcium release-activated calcium channel protein 1 (Orai1) and the STIM1 on ER/SR (Ong et al., 2007; Liao et al., 2008, 2009). Following the emptying of SR Ca^2+^ stores, STIM1 is activated and leads to the redistribution of Orai1 and TRPCs to assemble the SOCE channel complex in mediating the Ca^2+^ entry (Liao et al., 2009). Recent studies have also demonstrated TRPC channels may play important roles in the regulation of electromechanical activity of the developing heart (Sabourin et al., 2011), Ca^2+^ paradox injury (Kojima et al., 2010), as well as in pathological structural and functional remodeling after myocardial infarction (Makarewich et al., 2014). Hyperforin, an extract from the medicinal herb St. John’s Wort (*Hypericum perforatum*), exhibits antidepressant properties, although the underlying mechanism is not clear yet (Chatterjee et al., 1998). It has been shown that hyperforin is a selective activator of TRPC6, and leads to a non-selective cation current (Leuner et al., 2007). Therefore, we have used hyperforin as a tool to test potential deleterious effect of TRPC channel hyperactivation.

In the present study, we demonstrate the presence of SOCE in adult mouse ventricular myocytes, and elucidate the possible roles of SOCE/TRPCs in promoting cardiac arrhythmias via facilitating the generation of inward current and calcium load. Our findings support a missing link between upregulated SOCE/TRPCs activities, especially TRPC6, to lethal arrhythmias in the heart.

## Materials and Methods

All animal experimental procedures were reviewed and approved by the Institutional Animal Care and Use Committee at the Rutgers New Jersey Medical School. All chemicals were purchased from Sigma Aldrich unless indicated. The functional (pore inhibitory) antibodies for TRPC1, 3 and 6 were a generous gift from Dr. Robert M. Graham (University of Sydney, and Victor Chang Cardiac Research Institute). These antibodies recognize the putative pore-forming region of mouse TRPC channels specifically, and pre-incubation with anti-TRPC6 antibody could effectively block the rise of intracellular Ca^2+^ concentration ([Ca^2+^]) upon restoration of external calcium load (1.8 mM) (Mohl et al., 2011). The SERCA inhibitor thapsigargin (Tha), SOCE inhibitors ML-9, gadolinium and Pyr3, and TRPC6 activator hyperforin were dissolved in DMSO as stock solutions before diluting into the bath solution at the final concentrations. The maximum DMSO concentration was < 0.2% (vol/vol).

### Cell Isolation

Ventricular myocytes were enzymatically isolated from the left ventricles of FVB mice (male, 4–6 months). Briefly, hearts were removed from mice anesthetized with overdosed isoflurane, and were perfused retrogradely at 37°C in Langendorff fashion with nominally Ca^2+^-free Tyrode’s solution containing 1.0 mg/mL collagenase (Type II; Worthington) and 0.1 mg/mL thermolysin (Sigma) for 15 min. The hearts were removed from the perfusion apparatus after washing out the enzyme solution, the left ventricle were gently teased apart with forceps in a petri dish and the myocytes were filtered through a nylon mesh. The Ca^2+^ concentration was gradually increased to 1.0 mM, and the cells were stored at room temperature and used within 8 h (Xie and Weiss, 2009).

### Ca^2+^ Measurement

Myocytes were loaded with the Ca^2+^ indicator Fluo-4 AM by incubating them for ∼30 min in bath solution containing 4 μM Fluo-4 AM (Molecular Probes), after which the cells were washed and placed in a heated chamber on an inverted microscope. Fluo-4 fluorescence was excited at ∼485 nm and the emission was measured at ∼530 nm using a Nikon Eclipse TE200 inverted microscope with a Fluor x40 oil objective lens (numerical aperture 1.3). The fluorescence signals were recorded using an Andor Ixon charge-coupled device (CCD) camera (Andor Technology) operated with Imaging Workbench software (INDEC BioSystems) at 50 frames per second with a spatial resolution of 500 × 400 pixels. Fluorescence intensity was measured as the ratio of the fluorescence (*F*) over the basal diastolic fluorescence (*F_0_*). The spontaneous waves were quantified by counting the wave frequency (per minute) appeared after a 3-min perfusion of HF 0.1 μM, compared to the control state.

### Electrophysiological Recording

Myocytes were patch-clamped using the whole-cell configuration of the perforated patch-clamp technique (240 μg/ml amphotericin B). Voltage or current signals were measured with a MultiClamp 700A patch-clamp amplifier controlled by a personal computer using a Digidata 1322 acquisition board driven by pCLAMP 10 software (Molecular Devices, Sunnyvale, CA, United States). For AP recordings, patch pipettes (resistance 2–5 MΩ) were filled with internal solution containing (in mM): 110 K-aspartate, 30 KCl, 5 NaCl, 10 HEPES, 0.1 EGTA, 5 MgATP, 5 Na_2_-phosphocreatine, 0.05 cAMP, pH 7.2 with KOH. The cells were superfused with Tyrode’s solution containing (in mM): 136 NaCl, 4.0 KCl, 0.33 Na_2_PO_4_, 1.0 CaCl_2_, 1 MgCl_2_, 10 glucose and 10 HEPES, pH 7.4 adjusted with NaOH. APs were elicited with 2-ms, 2- to 4-nA square pulses at a PCL of 6 s.

The whole-cell I–V relationship was measured by applying ramp pulses from -110 to +50 mV and the holding potential was -80 mV. K^+^, L-type Ca^2+^, Na^+^-Ca^2+^ exchange currents were previously blocked with a K^+^-free extracellular solution with (in mM) 140 NaCl, 1.0 CaCl_2_, 0.5 MgCl_2_, 0.33 NaH_2_PO_4_, 5.5 glucose, 0.01 Nifedipine, 0.002 SEA400 and 5 HEPES ( pH 7.4 adjusted with NaOH), and a pipette solution containing (in mM) 90 Cs-aspartate, 30 CsCl, 20 traethylammonium chloride (TEA-Cl), 2 MgCl_2_, 5 Tris-ATP, 0.1 Li_2_-GTP, 5 EGTA, 2 CaCl_2_ and 5 HEPES (pH adjusted to 7.2 with CsOH)[17]. All patch clamp and Ca^2+^ imaging experiments were performed at 37°C.

### ECG Recording and Arrhythmia Induction Testing

(Pseudo-)Lead II ECGs were recorded in isolated Langendorff-perfused hearts. A pair of Ag-AgCl electrodes were placed close to the apex of the left ventricle as well as at the right atrial appendage to obtain ECG signals at a sampling rate of 1 kHz. Two additional platinum electrodes were placed on the free wall of the right ventricle for stimulation using a stimulator (Grass) triggered by a custom-designed computer program. To induce ventricular arrhythmias, a standard S_1_-S_2_ arrhythmia induction protocol at twice the pacing threshold intensity was adapted from Jeron et al. (2000). Following a 20-beat train with a basic cycle length of 100 ms (S_1_), 3 extra stimuli (S_2_) with a coupling cycle length of 50, 40, or 30 ms, respectively, were introduced. Each of these 3 sets of stimulation were repeated 3 times. Arrhythmias will be categorized into 5 groups and assigned the following points: 0 points, no arrhythmia; 1 point, 1–3 premature ventricular complexes (PVCs); 2 points, non-sustained VT (4–10 consecutive PVCs, including bigeminal/trigeminal PVCs); 3 points, sustained VT (>10 consecutive PVCs); and 4 points, VF/SCD (Zhao et al., 2018).

### Statistical Analysis

Data are presented as mean ± SEM, and at least 3 animals were used for each group. Statistical significance was assessed using paired, unpaired Student’s *t*-tests or ANOVA analysis, with *P* < 0.05 considered significant.

## Results

### SOCE Exists in Adult Cardiac Myocytes

**FIGURE 1:**
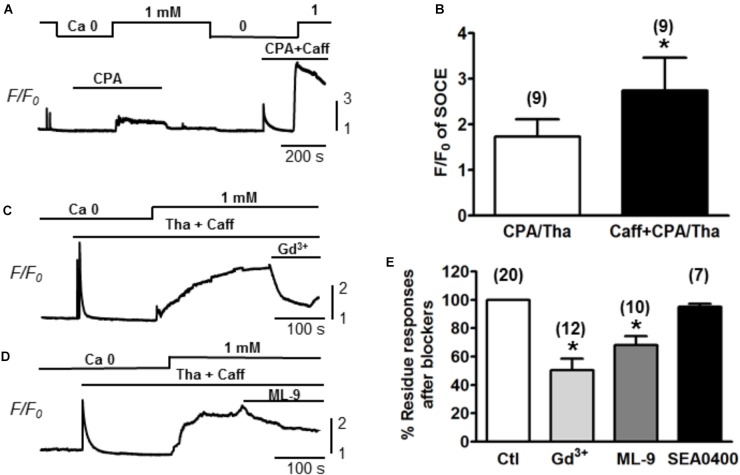
Store-operated Ca^2+^ entry measured in adult mouse ventricular myocytes. **(A)** A representative recording of SOCE from an adult mouse ventricular myocyte. Ca^2+^ fluorescence intensity (*F/F_0_*) in normal Tyrode’s solution (1 mM Ca^2+^) and Ca^2+^-free (Ca^2+^) Tyrode’s solution was measured. SOCE was detected under partial SR Ca depletion by 10 μM CPA (an inhibitor of SERCA) alone or complete depletion by 10 μM CPA and 10 mM caffeine (CPA + Caff). **(B)** Summary of *F/F_0_* responses (SOCE) recorded in the presence of 10 μM CPA or 1 μM thapsigargin (Tha, another SERCA blocker) (CPA/Tha) alone (1.7 ± 0.4) or together with caffeine (2.7 ± 0.7, ^∗^*p* < 0.05), suggesting the complete depletion of SR Ca is required for maximal SOCE activation. **(C,D)** Representative traces of SOCE and its inhibition by TRPC or SOCE blockers (i.e., Gd^3+^ and ML-9). **(E)** Summary data demonstrating the putative SOCE was inhibited by SOCE/TRPC channel blockers (39.8 ± 4.5% inhibition by 1 mM Gd^3+^ and 31.8 ± 6.3% inhibition by 10 μM ML-9. ^∗^*p* < 0.05 compared to control, Student’s *t*-Test) but not SEA0400 (4.9 ± 2.3%).

Ventricular myocytes were isolated from adult mouse hearts and were loaded with Fluo-4 AM for measurement of Ca^2+^. The changes of Ca^2+^ level (reflected by Fluo-4 fluorescence intensity) were measured by increasing extracellular Ca^2+^ concentration ([Ca^2+^]) from 0 to 1 mM (Correll et al., 2015). SOCE was traditionally initiated by emptying SR stores with Tha or CPA (Ong et al., 2007). Both Tha and CPA are SERCA blockers, which are able to passively deplete the SR by inhibiting the SR Ca^2+^ up-taking from the cytosol. A typical protocol for inducing SOCE is demonstrated in Figure 1A. Following the SR depletion by using 10 μM CPA, a moderate increase of Ca^2+^ level (as showed by F/F_0_ elevation) was observed when [Ca^2+^] was changed from 0 to 1 mM. In order to maximally/completely deplete SR Ca^2+^, in addition to CPA, we also employed 10 mM caffeine to fully open RyR. As a result, a much larger elevation of Ca^2+^ level was induced when [Ca^2+^] was changed from 0 to 1 mM (Figure 1A). The same phenomena were observed when caffeine was combined with 1 mM Tha. We therefore defined the maximal SOCE amplitude to be the elevation of Ca^2+^ level after the SR Ca^2+^ was maximally depleted by using caffeine in addition to CPA or Tha (Caff + CPA/Tha). As shown in Figure 1B, the amplitude of SOCE obtained after caffeine (10 mM) + Tha (1 μM)/CPA (10 μM) (F/F_0_ = 2.7 ± 0.7) was markedly higher than that after Tha/CPA only (F/F_0_ = 1.7 ± 0.4, *n* = 9, ^∗^*p* < 0.05), suggesting the existence of SOCE in adult cardiac myocytes, and a maximal SOCE activation requires the complete depletion of SR Ca^2+^. This SOCE was effectively blocked by SOCE/TRPC blockers gadolinium (Gd^3+^, inhibited by 39.8 ± 4.5%, *n* = 12, ^∗^*p* < 0.05) and ML-9 (inhibited by 31.8 ± 6.3 %, *n* = 10, ^∗^*p* < 0.05 respectively), but not by Na^+^/Ca^2+^ exchanger (NCX) inhibitor SEA0400 (by 4.9 ± 2.3%, p > 0.05; *n* = 7, Figures 1C,D).

### TRPC Channels Contribute to the Function of SOCE in Adult Myocytes

**FIGURE 2:**
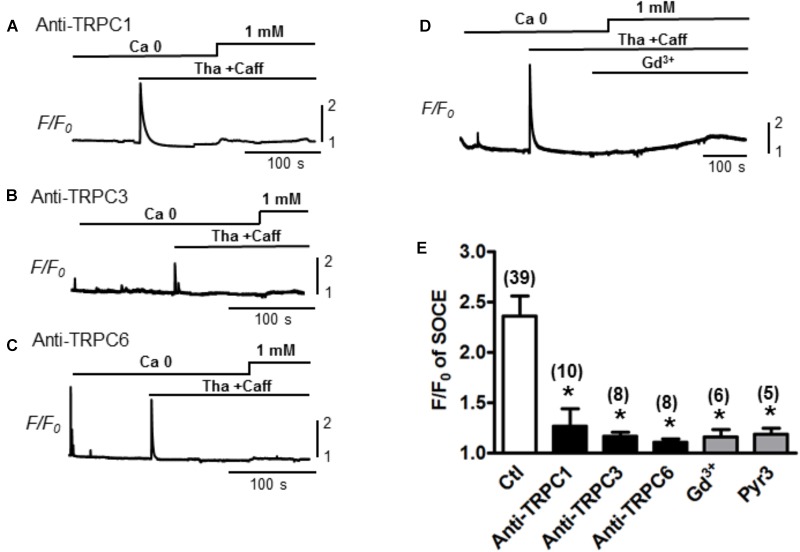
Store-operated Ca^2+^ entry inhibition by functional antibodies or inhibitors of TRPCs. **(A–C)** Representative traces of Ca^2+^ fluorescence (*F/F_0_*) recorded from myocytes pre-treated with TRPC channel antibodies for 30 min before recording. SOCEs were evaluated using a standard protocol (i.e., 0 Ca + Tha + Caff). **(D)** A representative trace of SOCE after pretreatment with Gd^3+^ 1 mM for 100 s. **(E)** A bar graph summarizing the effects of the TRPC antibodies and Gd^3+^ on SOCE (Ctl: 2.4 ± 0.2, TRPC1 Ab: 1.4 ± 0.2, TRPC3 Ab:1.2 ± 0.2, TRPC6 Ab: 1.1 ± 0.1 and Gd^3+^:1.2 ± 0.1, Pyr3: 1.2 ± 0.1, Student’s *t*-Test), suggesting TRPC channels account for SOCE.

To determine whether the TRPC channels contribute to the modulation of SOCE in adult cardiac myocytes, functional (pore inhibitory) antibodies for TRPC1, 3 and 6 were added, respectively, to pretreat the myocytes for 30 min before SOCE was evaluated following the standard protocol with complete depletion of SR Ca^2+^ by Tha (1 μM) + caffeine (10 mM). Control myocytes without pretreatment with antibodies exhibited a peak SOCE amplitude of F/F_0_ = 2.4 ± 0.2, *n* = 39, whereas all three TRPC1, 3 or 6 antibodies significantly inhibited the peak of SOCE to F/F_0_ = 1.4 ± 0.2 (*n* = 10), 1.2 ± 0.2 (*n* = 8), 1.1 ± 0.1 (*n* = 8), respectively (Figure 2, ^∗^*p* < 0.05). Similar inhibitory effects to 1.2 ± 0.1 (*n* = 5) by Gd^3+^ or to 1.2 ± 0.1 (*n* = 6) by the TRPC3 blocker Pyr3 pre-perfused in the bath for 10–15 min were also observed (^∗^*p* < 0.05, respectively) (Figure 2). These results suggest that various TRPC channel subtypes may account for the SOCE generation in adult myocytes.

### Hyperforin Potentiates the SOCE in Adult Myocytes

**FIGURE 3:**
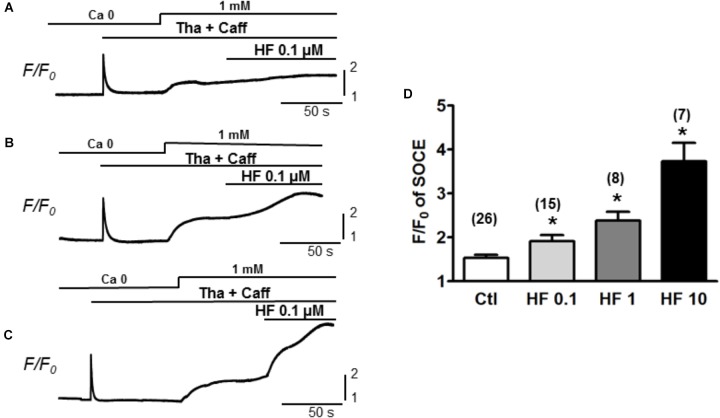
TRPC6 activator hyperforin potentiates the SOCE in adult mouse cardiac myocytes. **(A–C)** Representative recordings of SOCE from adult mouse myocytes. Ca^2+^ fluorescence intensity (F/F_0_) in normal Tyrode’s solution (1 mM Ca^2+^) and Ca^2+^-free (0 Ca) Tyrode’s solution was measured. SOCE was detected under complete depletion by 0.1 μM thapsigargin and 1 mM caffeine (Tha + Caff), and was further enhanced by hyperforin (HF) at different concentrations. **(D)** Summary of *F/F_0_* responses (SOCE) in the presence of 0.1, 1, and 10 μM hyperforin, which potentiated SOCE in a concentration-dependent fashion. (^∗^*p* < 0.05 compared to control, Student’s *t*-Test).

Furthermore, the participation of TRPC6 in SOCE was confirmed by using various concentrations of hyperforin, a potent activator of TRPC6 (Leuner et al., 2007). As shown in Figure 3, SOCE was partially activated by using moderate concentration of caffeine (1 mM) + Tha (0.1 μM) (F/F_0_ = 1.5 ± 0.1, *n =* 26). Hyperforin further potentiated the SOCE level in a concentration-dependent manner in adult mouse ventricular myocytes. The amplitudes (F/F_0_) of SOCE in the presence of hyperforin at 0.1, 1, and 10 μM were, 1.9 ± 0.1 (*n* = 15), 2.4 ± 0.2 (*n* = 8) and 3.7 ± 0.4 (*n* = 7), respectively (Figure 3D, ^∗^*p* < 0.05), which were all significantly higher than the control SOCE value before hyperforin application.

### Activation of TRPC by Hyperforin Increases the Calcium Load in Mouse Cardiac Myocytes

**FIGURE 4:**
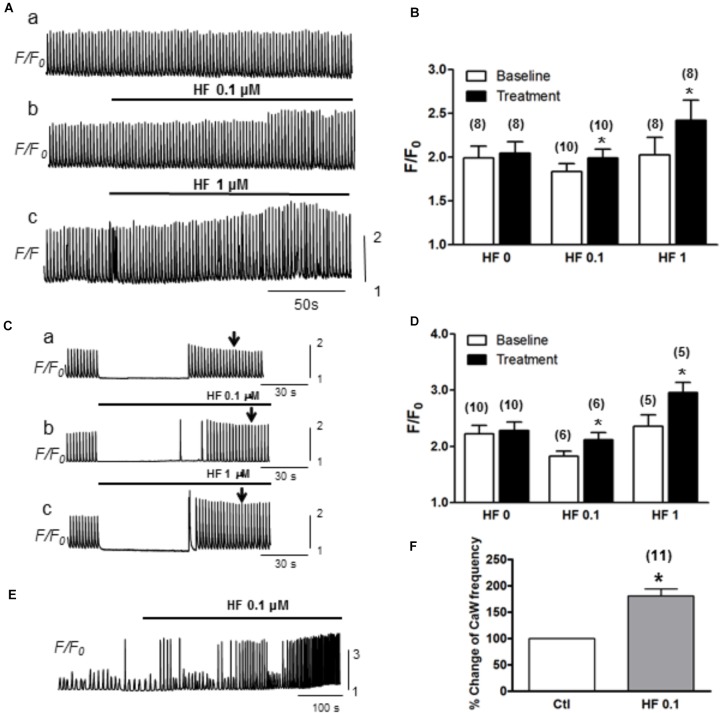
Hyperforin enhances CaT amplitude. **(A)** A representative trace of CaTs facilitated by hyperforin (0.1 and 1 μM) when the cell was paced at a pacing cycle length (PCL) of 2 s. **(B)** Summary of the amplitudes of CaTs potentiated by hyperforin. *F/F_0_* increased from 1.9 ± 0.1 to 2.1 ± 0.1, *n* = 10 at 0.1 μM and from 2.0 ± 0.2 to 2.4 ± 0.1, *n* = 8 at 1 μM hyperforin, respectively (^∗^*p* < 0.05). **(C)** Representative traces of calcium transients were measured before and after a 90s-perfusion of hyperforin (0.1 – 1 μM) while the cells were kept quiescent. **(D)** Summary of calcium transient amplitude by hyperforin perfusion. **(E)** A representative trace of Ca^2+^ waves (with [Ca^2+^] 4 mM) facilitated by hyperforin (0.1 μM). **(F)** Summary of the changes in Ca^2+^ wave frequencies caused by hyperforin, which promotes the Ca^2+^ waves by increasing the frequency to 180.5 ± 13.5% (*n* = 11, ^∗^*p* < 0.05 vs. control).

Next, we evaluated how hyperforin-activated TRPC may affect Ca^2+^ transients and Ca^2+^ waves. Ca^2+^ transients were measured by pacing myocytes continuously at a PCL of 2 s. As shown in a representative trace (Figure 4A), the amplitudes of Ca^2+^ transients were markedly enhanced by the application of hyperforin at concentrations of 0.1 μM (Figure 4Aa) and 1 μM (Figure 4Ab). Summarized data demonstrated that F/F_0_ was increased from 1.9 ± 0.1 to 2.1 ± 0.1 (^∗^*p* < 0.05, *n* = 10) by 0.1 μM hyperforin, and from 2.0 ± 0.2 to 2.4 ± 0.1 (^∗^*p* < 0.05, *n* = 8) by 1 μM hyperforin. To exclude the possibility that the enhancement of Ca^2+^ transients might be due to the potentiation any other voltage-gated Ca influx mechanisms, we then used a different protocol in which the cells were kept quiescent when they were perfused with hyperforin (0.1 or 1 μM) for 1 min (Figure 4C) (the field stimulation was terminated prior to hyperforin addition and then restarted 1 min later). To exclude any influence of post-rest potentiation and subsequent decay (i.e., negative staircase phenomenon), the amplitude of Ca^2+^ transient were measured when it reached a steady state level (∼30 s after the restart of stimulation) as indicated by the arrow in each panel). We found that Ca^2+^ transient amplitude was enhanced by hyperforin treatment even the cells remained in resting condition. Ca^2+^ transient amplitudes increased from 1.8 ± 0.2 to 2.1 ± 0.3 (*n* = 6, ^∗^*p* < 0.05) by 0.1 μM hyperforin and from 2.4 ± 0.5 to 3.0 ± 0.4 (^∗^*p* < 0.05, *n* = 5) by 1 μM hyperforin (Figure 4D), in comparison to the control group (no hyperforin treatment, from 2.2 ± 0.5 to 2.3 ± 0.4, *n* = 9). These data suggest that hyperforin promoted Ca^2+^ entry through sarcolemmal membrane and increase of cellular/SR Ca^2+^ load, unlikely via voltage-gated Ca^2+^ channels.

Next, we further assessed the effect of hyperforin on spontaneous Ca^2+^ waves. In this experiment, ventricular myocytes were perfused with Tyrode’s solution containing higher [Ca^2+^] (4 mM) without being stimulated. As shown in Figure 4E, spontaneous Ca^2+^ waves were observed under baseline in normal cells (Zhao et al., 2013). These spontaneous Ca^2+^ waves were significantly facilitated by hyperforin (0.1 μM) with a frequency increase from 22 ± 3/min under control condition to 38 ± 4/min after hyperforin treatment (Figures 4E,F, *n* = 11, ^∗^*p* < 0.05). Taken together, the results shown in Figures 3, 4 have suggested that hyperforin is able to activate both SOCE under SR Ca^2+^ depletion condition and TRPC6 channels independent of SR Ca^2+^ depletion.

### Significant Inward Currents Are Induced by SR Depletion or by Hyperforin

**FIGURE 5:**
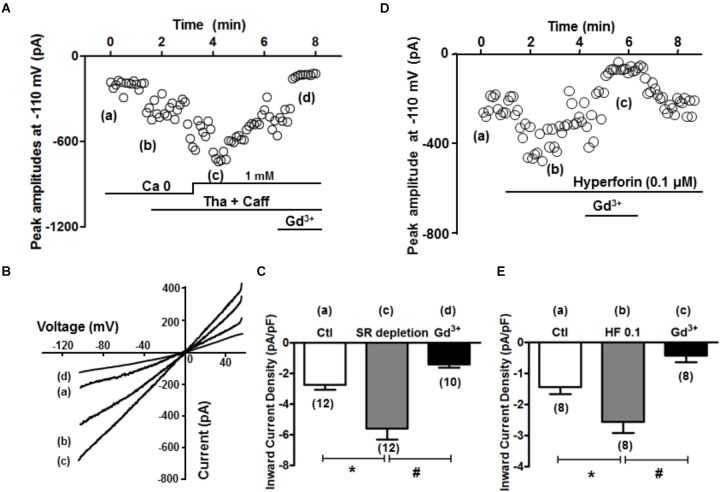
SR depletion and the presence of hyperforin induced significant inward current. **(A)** A representative plot of currents measured at –110 mV over time. The same treatment protocol to deplete SR Ca^2+^ (Tha + Caff) was used. **(B)** I–V curves at time points a–d (control, calcium free, after SR depletion, and in the presence of Gd^3+^, respectively) indicated in **(A)**. **(C)** Summary of the inward current densities at –110 mV measured under control and after SR depletion (^∗^*p* < 0.05, Student’s *t*-Test). **(D)** A representative plot of currents measured at –110 mV over time, and the presence of hyperforin induced significant inward current entry. **(E)** Summary of the inward current densities at –110 mV measured under control and after SR depletion. Hyperforin (0.1 μM) induced a significant increase of inward current (from –1.4 ± 0.2 pA/pF to –2.6 ± 0.4 pA/pF, *n* = 8, ^∗^*p* < 0.05), which was then inhibited by the SOCE blocker Gd^3+^ (1 mM) (–0.4 ± 0.2 pA/pF, *n* = 8, ^∗^*p* < 0.05, Student’s *t*-Test).

The inward current when SOCE occurred (i.e., SOCE current, I_OCE_) after SR Ca^2+^ depletion was subsequently measured. As described in the Method section, K^+^, Na^+^, L-type Ca, and Na^+^-Ca^2+^ exchange currents were pre-blocked. Whole-cell currents were recorded under the voltage clamp condition using a ramp protocol from -110 to +50 mV, and the inward current amplitudes at -110 mV were evaluated. As shown in time course and representative traces (Figures 5A,B), I_OCE_ was induced when [Ca^2+^] was changed from 0 to 1 mM after SR depletion (from point a to point c). As summarized in Figure 5C, we observed a marked increase from -2.7 ± 0.3 to -5.6 ± 0.7 pA/pF (*n* = 12 each, ^∗^*p* < 0.01) in the inward current, which was attenuated by the application of 1 mM Gd^3+^ (-1.4 ± 0.2 pA/pF, *n* = 10, ^∗^*p* < 0.01). The I/V relationship revealed a reversal potential around 0 mV, indicating its non-selective property, which is consistent with TRPC channels as reported by Kojima et al. (2012). As shown in Figures 5D,E, hyperforin (0.1 μM) also significantly enhanced the inward current from -1.4 ± 0.2 to -2.6 ± 0.4 pA/pF (*n* = 8, ^∗^*p* < 0.05 compared to control), which was effectively blocked by 1 mM Gd^3+^ (-0.4 ± 0.2 pA/pF, *n* = 8, #*p* < 0.05 compared to hyperforin).

### Activation of SOCE or TRPC6 Contributes to Cardiac Arrhythmogenesis

**FIGURE 6:**
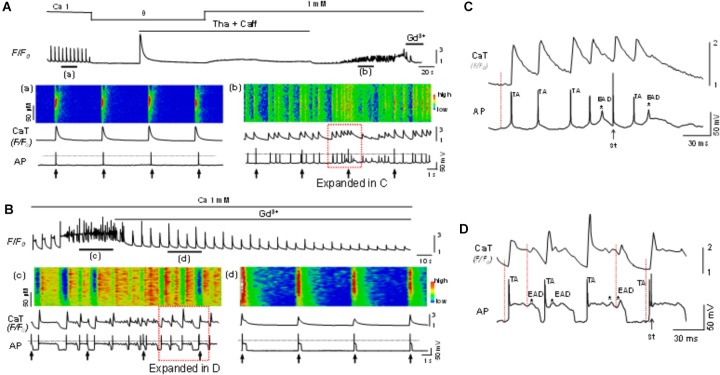
Store-operated calcium entry contributes to cellular arrhythmogenesis. **(A)** Top panel: A Ca^2+^ fluorescence (*F/F_0_*) recording. **(a,b)**: Simultaneous recordings of linescan image, whole-cell calcium fluorescence (*F/F_0_*) and action potentials (AP) at the time windows a and b indicated in the top panel. The cell was paced at a pacing cycle length (PCL) of 6 s (indicated by the arrows underneath the AP trace). EADs, DADs and triggered APs appeared after SOCE was activated, indicating SOCE contributes to the generation of arrhythmias. **(B)** In another cell, the same protocol as in A was used to activate SOCE. The SOCE-induced EADs (indicated by the asterisks), DADs, and triggered APs **(c)** were eliminated by Gd^3+^ (1 mM) **(d)**. The 0 mV level is indicated by the dotted line in each panel. **(C)** Expanded region within the red box as indicated in **(Ab)**. **(D)** Expanded region within the red box as indicated in **(Bc)**. Note initiation (indicated by the dotted vertical lines) of the spontaneous depolarization phase (DADs triggering action potential) and occurrence of EADs (indicated by ^∗^) preceded the elevation of intracellular Ca^2+^. TA, triggered action potential; St, stimulation.

The potential role of SOCE in arrhythmogenesis at cellular level was studied by simultaneous recording of whole-cell Ca^2+^ imaging and membrane potential. Normal APs were recorded when myocytes were paced at a PCL of 6 s (Figure 6Aa). The cell was then exposed to the standard protocol to activate SOCE. EADs, DADs and TAs, as key indicators for cellular arrhythmias, appeared after the activation of SOCE and removal of SR uptake and release inhibition (Figures 6Ab, 6Bc). In order to establish a connection between membrane potential changes and corresponding changes in intracellular Ca^2+^, we examined temporal correlation between them by comparing the onsets of spontaneous depolarizations (i.e., DAD) of membrane potential and corresponding elevation of intracellular Ca^2+^. As shown in the expanded regions (Figures 6C,D) of the boxed periods in panel Figures 6Ab,Bc, the initiation (indicated by the dotted vertical lines) of the spontaneous depolarizations (DADs triggering APs) always preceded the elevation of intracellular Ca^2+^, suggesting a predominant inward current, presumably via TRPC channels, contributes to the arrhythmogenic membrane depolarization. Although the SR uptake and release inhibition (Tha + Caff) had been removed in this condition when arrhythmias occurred, it seemed that Ca^2+^ release and NCX current did not play a causal role in spontaneous membrane depolarization, in which case the initiation of intracellular Ca^2+^ elevation should occur earlier than membrane potential changes. This relation has been well discussed in our previous publication (Zhao et al., 2012). The EADs, DADs and TAs were eliminated by Gd^3+^ treatment, although the action potential duration (APD) remained longer compared to control (Figure 6Bd vs. Figure 6Aa). SOCE-induced EADs, DADs and TAs were observed in 10 out of 12 cells in total. Such arrhythmic events were not seen before the cells were treated with Tha + Caff to deplete SR Ca^2+^ (Figure 6Aa). These data suggested the contribution of SOCE to the generation of arrhythmias at cellular level.

**FIGURE 7:**
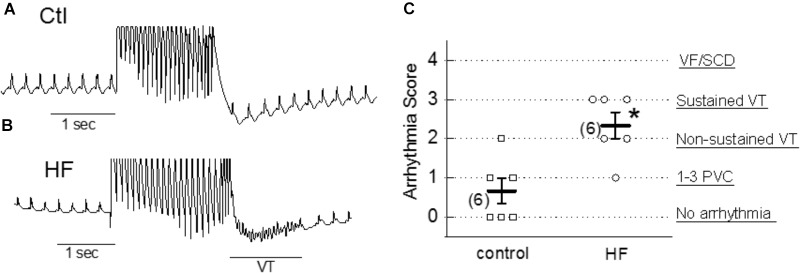
Proarrhythmic effect of hyperforin in *ex vivo* hearts. **(A,B)** Pseudo-Lead II ECG signals recorded from control (ctl) and 20 min after perfusion with 100 nM hyperforin (HF). Programmed S1–S2 stimulations were applied to evaluate the vulnerability to arrhythmias in each case. Sustained VT was observed in HF-treated heart. **(C)** Summarized arrhythmia scores for control and hyperforin groups, ^∗^*p* < 0.05, compared to control.

To examine the arrhythmogenic effect of activation of TRPC6 channel by hyperforin at the whole-heart level, hyperforin (0.1 μM) was perfused in *ex vivo* mouse hearts via a Langendorff perfusion apparatus for 20 min. Programmed stimulation protocols (as described in Section “Materials and Methods”) were used to assess the propensity to develop arrhythmias in each group. As shown in Figures 7A–C, hyperforin-perfused hearts were particularly susceptible to various types of arrhythmias and had an average arrhythmia score of 2.3 ± 0.3 (*n* = 6), which was significantly higher than hearts without treatment with hyperforin (0.7 ± 0.3, *n* = 6, *p* < 0.05).

## Discussion

### SOCE Exists in Adult Cardiac Myocytes

Store-operated calcium entry has been identified as a cellular mechanism that plays a fundamental role in Ca^2+^ signaling. In addition to replenish the SR stores, its long-term and immediate physiological significances have been profoundly explored in previous studies, includes cell proliferation, apoptosis, muscle contraction and intracellular Ca^2+^ oscillations (see (Parekh and Putney, 2005) for review). Although it was primarily considered important only for non-excitable cells, growing evidence has demonstrated a crucial role of I_OCE_ in central nervous system (Baba et al., 2003), aortic myocytes (Trepakova et al., 2001) and muscle cells (Hopf et al., 1996; Kurebayashi and Ogawa, 2001; Shin et al., 2003). Due to the prevalence of distributions of L-type Ca^2+^ channel, NCX, RyR and SR Ca^2+^-ATPase 2 (SERCA2) (Bers, 2008), the roles of SOCE in cardiomyocytes have been overlooked for balancing the Ca^2+^ loading. Until recently, capacitative calcium entry was observed in adult rat cardiomyocyte (Hunton et al., 2004), and the modulation effects of SOCE in neonatal or adult hypertrophic mice cardiomyocytes were demonstrated (Hunton et al., 2002; Voelkers et al., 2010; Wu et al., 2010).

Our present study demonstrated that SOCE and I_OCE_ exist in normal adult ventricular myocytes subsequent to a complete depletion of SR Ca^2+^ storage. SR Ca^2+^ level can be physiologically reduced following the activation of calcium-releasing signals mediated by IP3R or RyR. Whereas under our experimental condition, specific inhibitors of SR Ca^2+^ pump (i.e., Tha or CPA) are usually employed to induce SOCE, previous studies seemed to produce only moderate increase in normal adult cardiac myocytes when exposed to CPA or Tha separately (Hunton et al., 2004; Wu et al., 2010). Similarly, Kojima et al also demonstrated the SOCE activity in the mice ventricular myocytes by combining the application of angiotensin II and Tha to deplete the SR store (Kojima et al., 2012). Alternatively, caffeine as potent RyR activator has also been shown to be effective to initiate SOCE (Albert and Large, 2002; Kojima et al., 2010). In the present study, we applied caffeine together with Tha or CPA to obtain a maximum activation of SOCE, therefore, adding indisputable evidence supporting the presence of SOCE in adult mouse ventricular myocytes. As shown in the results, the combination of caffeine and SERCA inhibitors (Tha or CPA) effectively potentiated SOCE to a larger extent (F/F_0_: over 2.5) compared to that induced only by SERCA inhibitor. This Ca^2+^ entry can be effectively inhibited by acute application of SOCE blockers Gd^3+^ and ML-9, but not by the NCX inhibitor SEA0400. We have noticed that neither Gd^3+^ (also blocks L-type Ca^2+^ channel and mechano-sensitive ion channel) nor ML-9 (also inhibits myosin light chain kinase) is completely selective to TRPC channels. However, our experiments using selective antibodies of TRPC channels provide more convincing evidence for the involvement of TRPC channels.

It should be pointed out that caffeine also functions as a PDE inhibitor (Rohrig et al., 2017), which increases cAMP concentration and PKA activation at cellular level. Therefore, we cannot completely exclude the possibility that caffeine-induced enhancement of SOCE may be partially attributed to SR Ca^2+^ load-independent pathway, e.g., PKA-dependent activation. This issue needs to be further elucidated.

### Contribution of TRPCs to SOCE

Due to the absence of Ca^2+^ selectivity, the involvement of TRPCs in SOCE is still under debate. For example, the expression of TRPC3 has been examined in DT40 B lympohocytes by two groups individually. One group demonstrated the TRPC3 activation is triggered in response to PLC-γ or PLC-β enzymes, but independent of SR depletion (Venkatachalam et al., 2001). Meanwhile, the other group showed substantial greater divalent cation entries by heterologous expression of hTRPCs in IP3R-knockout cells (Vazquez et al., 2001). A subsequent study revealed that these different regulation mechanisms are determined by the different expression levels of TRPC3. At low expression level, TRPC3 tend to form store-operated channels, while at higher expression level, it forms receptor-activated channels which is insensitive to Gd^3+^ (Vazquez et al., 2003). Above evidence implicated a crucial role of TRPC stoichiometry in determining its function during the subunit assembling process. It seems that low expression level of TRPCs may be insufficient to yield functional TRP complex, but may prompt the formation of a heteromeric Orai/TRPC model for SOCE. Although the participation of TRPC channels in a STIM1-stimulated Orai/TRPC model for SOCE is still under debate (Parekh and Putney, 2005; DeHaven et al., 2009), a collection of studies has indicated the roles of TRPC family in SOCE (Liao et al., 2008, 2009). Our present data provide further evidence that revealed the contribution of TRPC channels to SOCE generated in adult mouse ventricular myocytes. As showed in Figures 1–3, SOCE was markedly attenuated by a perfusion with TRPC blockers Gd^3+^ and Pyr3. Since Gd^3+^ and Pyr3 are considered less selective, we also used selective TRPC pore inhibitory antibodies. When the pore-blocking antibodies for TRPC 1, 3 and 6 were employed (Figure 2), they each resulted in partial inhibition on SOCE. In addition, the selective TRPC6 activator hyperforin enhanced SOCE level, further implicating the role of TRPC6 in mediating store-operated calcium influxes. Significant expressions of TRPC1, 3 and 6 isoforms in mouse cardiomyocytes have been reported (Katayama et al., 1990; Mohl et al., 2011). In consistence with our observations, the SOCE level is potentiated in cardiac-specific TRPC3 expressing mice (Katayama et al., 1990), and is reduced in myocytes isolated from dominant-negative TRPC3, 4 and 6 transgenic mice (Wu et al., 2010). These results imply the TRPC channels may at least partially mediate the SOCE by forming either homo- or hetero-multimers. The combination of using functional (pore inhibitory) antibodies for TRPCs and a range of different TRPC and SOCE blockers/activators, revealed that TRPC channels account for the prominent SOCE after SR depletion in adult myocytes.

### Roles of SOCE/TRPCs in Arrhythmogenesis

Transient receptor potential canonical channels carry inward currents, therefore may modulate cardiac rhythm and exert potential arrhythmic effects (Hirose et al., 2011; Sabourin et al., 2011). SOCE mediated proarrhythmic effects have been suggested in different models. For example, overexpression of STIM, which is an important sensor for SOCE communicating between the sarcolemma and SR, results in leaky RyR and spontaneous Ca^2+^ transients (Correll et al., 2015). The upregulated expression of TRPC3/4 induced a proarrhythmic SOCE-like activity in adult rat ventricular cardiomyocytes (Domínguez-Rodríguez et al., 2015). A study on the developing chick heart has also demonstrated that the cardiac arrhythmias modulated by the activation of SOCE (Sabourin et al., 2011). The heart rate measured was dramatically increased by SERCA inhibitor CPA within 5 min, and this CPA-induced tachycardia was prevented by a treatment with TRPCs blocker SKF-96265 effectively. Subsequent to the SR depletion by activation of RyR with caffeine and by inhibition of SERCA with Tha, substantial Ca^2+^ current influx was measured in our experimental setting, suggesting the TRPC channels act as store-operated channels in mediating inward depolarizing Ca^2+^ current. We observed the increase of inward current and recorded EADs/DADs/TAs and Ca^2+^ waves simultaneously after SOCE activation in most cases, indicating SOCE may account for the generation of arrhythmias in the adult cardiac myocyte. Gd^3+^ is the most potent TRPC blocker that effectively eliminates arrhythmic events induced by SOCE, which implies a therapeutic potential of Gd^3+^ for the arrhythmias induced by TRPC channel activation or SOCE.

### Hyperforin Exhibits Arrhythmogenic Effect

Hyperforin is known as an antidepressant compound extracted from St. John’s wort (*Hypericum perforatum*), and it was reported to selectively activate TRPC6 channels. Our data clearly demonstrated that hyperforin at concentrations of 0.1 and 1 μM predominantly potentiated the SOCE and I_OCE_ in adult mouse ventricular myocytes. It also facilitated the spontaneous Ca^2+^ waves and promoted the calcium transient amplitudes. A previous study has shown that hyperforin exerts complex actions on cortical neurons. In addition to promoting TRPC6 channels in plasma membrane, hyperforin may exert protonophore-like effect, triggering the release of Ca^2+^ and Zn^2+^ from mitochondria (Tu et al., 2010). However, in mouse ventricular myocytes, we did not observe the same effect of hyperforin (at 1 or 10 μM) to depolarize the mitochondrial membrane potential (as indicated by TMRM), while the protonophore FCCP dissipated mitochondrial membrane potential (data not shown). These results exclude the possibility that hyperforin induces Ca^2+^ release from internal store (i.e., mitochondria) in the setting of our experiments, but imply that hyperforin gives rise to Ca^2+^ most likely via activation of TRPC6 in cardiac myocytes. Our results have demonstrated that although the activation of SOCE requires SR Ca^2+^ depletion, its potential composing entity, TRPC channels, are able to be activated by HF independent of SR Ca^2+^ depletion (Figure 4). This is possible since others have reported TRPC channels may be activated under certain conditions (e.g., myocardial infarction) when SR is not necessarily depleted (Makarewich et al., 2014). We observed that perfusion with hyperforin could increase the arrhythmia score in *ex vivo* hearts, implying a possible arrhythmogenic role of SOCE/TRPC6 in the whole heart setting. TRPC6 channel was found to be important in the pathologic cardiac remodeling as a key component of a calcium-dependent regulatory loop (Kuwahara et al., 2006). Herein, our present study provides the first evidence that links hyperforin and TRPC6 activation to the enhanced susceptibility to arrhythmias. These data suggest an underlying mechanism for one of the side effects of St. John’s wort (hyperforin), which is heart palpitations, and caution is required when treating depression in patients who also have heart diseases.

## Author Contributions

HW, ZZ, NF, and L-HX performed the experiments and analyzed the data. HW and L-HX conceived and designed the research, interpreted results of experiments, prepared figures, and wrote manuscript. HW, ZZ, NF, and L-HX approved final version of manuscript.

## Conflict of Interest Statement

The authors declare that the research was conducted in the absence of any commercial or financial relationships that could be construed as a potential conflict of interest.

## Abbreviations

AP: action potential
CPA: cyclopiazonic acid
DAD: delayed afterdepolarization
EAD: early afterdepolarization
ER: endoplasmic reticulum
IP_3_R: IP_3_ receptor
NCX: sodium calcium exchanger
PCL: pacing cycle length
RyR: ryanodine receptor
SERCA: sarcoplasmic reticulum calcium ATPase
SOCE: store-operated calcium entry
SR: sarcoplasmic reticulum
STIM1: calcium sensor identified stromal interaction molecule 1
TAs: triggered activities
TRPC: transient receptor potential canonical

## References

[B1] Albert A. P. Large W. A. (2002). A Ca2+-permeable non-selective cation channel activated by depletion of internal Ca2+ stores in single rabbit portal vein myocytes. *J. Physiol.* 538(Pt 3) 717–728. 10.1113/jphysiol.2001.013101 11826160 PMC2290110

[B2] Baba A. Yasui T. Fujisawa S. Yamada R. X. Yamada M. K. Nishiyama N. (2003). Activity-evoked capacitative Ca2+ entry: implications in synaptic plasticity. *J. Neurosci.* 23 7737–7741. 10.1523/JNEUROSCI.23-21-07737.2003 12944501 PMC6740588

[B3] Berridge M. J. (1993). Inositol trisphosphate and calcium signalling. *Nature* 361 315–325. 10.1038/361315a0 8381210

[B4] Bers D. M. (2008). Calcium cycling and signaling in cardiac myocytes. *Annu. Rev. Physiol.* 70 23–49. 10.1146/annurev.physiol.70.113006.100455 17988210

[B5] Carafoli E. (2002). Calcium signaling: a tale for all seasons. *Proc. Natl. Acad. Sci. U.S.A.* 99 1115–1122. 10.1073/pnas.032427999 11830654 PMC122154

[B6] Chatterjee S. S. Bhattacharya S. K. Wonnemann M. Singer A. Muller W. E. (1998). Hyperforin as a possible antidepressant component of hypericum extracts. *Life Sci.* 63 499–510. 10.1016/S0024-3205(98)00299-9 9718074

[B7] Correll R. N. Goonasekera S. A. van Berlo J. H. Burr A. R. Accornero F. Zhang H. (2015). STIM1 elevation in the heart results in aberrant Ca2 handling and cardiomyopathy. *J. Mol. Cell Cardiol.* 87 38–47. 10.1016/j.yjmcc.2015.07.032 26241845 PMC4637225

[B8] DeHaven W. I. Jones B. F. Petranka J. G. Smyth J. T. Tomita T. Bird G. S. (2009). TRPC channels function independently of STIM1 and Orai1. *J. Physiol.* 587(Pt 10) 2275–2298. 10.1113/jphysiol.2009.170431 19332491 PMC2697298

[B9] Domínguez-Rodríguez A. Ruiz-Hurtado G. Sabourin J. Gómez A. M. Alvarez J. L. Benitah J. P. (2015). Proarrhythmic effect of sustained EPAC activation on TRPC3/4 in rat ventricular cardiomyocytes. *J. Mol. Cell Cardiol.* 87 74–78. 10.1016/j.yjmcc.2015.07.002 26219954

[B10] Hirose M. Takeishi Y. Niizeki T. Nakada T. Shimojo H. Kashihara T. (2011). Diacylglycerol kinase zeta inhibits ventricular tachyarrhythmias in a mouse model of heart failure. *Circ. J.* 75 2333–2342. 10.1253/circj.CJ-10-1213 21778596

[B11] Hopf F. W. Reddy P. Hong J. Steinhardt R. A. (1996). A capacitative calcium current in cultured skeletal muscle cells is mediated by the calcium-specific leak channel and inhibited by dihydropyridine compounds. *J. Biol. Chem.* 271 22358–22367. 10.1074/jbc.271.37.22358 8798397

[B12] Huang J. van Breemen C. Kuo K. H. Hove-Madsen L. Tibbits G. F. (2006). Store-operated Ca2+ entry modulates sarcoplasmic reticulum Ca2+ loading in neonatal rabbit cardiac ventricular myocytes. *Am. J. Physiol. Cell Physiol.* 290 C1572–C1582. 10.1152/ajpcell.00226.2005 16421209

[B13] Hunton D. L. Lucchesi P. A. Pang Y. Cheng X. Dell’Italia L. J. Marchase R. B. (2002). Capacitative calcium entry contributes to nuclear factor of activated T-cells nuclear translocation and hypertrophy in cardiomyocytes. *J. Biol. Chem.* 277 14266–14273. 10.1074/jbc.M107167200 11827959

[B14] Hunton D. L. Zou L. Pang Y. Marchase R. B. (2004). Adult rat cardiomyocytes exhibit capacitative calcium entry. *Am. J. Physiol. Heart Circ. Physiol.* 286 H1124–H1132. 10.1152/ajpheart.00162.2003 14630640

[B15] Jeron A. Mitchell G. F. Zhou J. Murata M. London B. Buckett P. (2000). Inducible polymorphic ventricular tachyarrhythmias in a transgenic mouse model with a long Q-T phenotype. *Am. J. Physiol. Heart Circ. Physiol.* 278 H1891–H1898. 10.1152/ajpheart.2000.278.6.H1891 10843886

[B16] Katayama Y. Shimizu J. Suzuki S. Memezawa H. Kashiwagi F. Kamiya T. (1990). Role of arachidonic acid metabolism on ischemic brain edema and metabolism. *Adv. Neurol.* 52 105–108. 2118711

[B17] Kojima A. Kitagawa H. Omatsu-Kanbe M. Matsuura H. Nosaka S. (2010). Ca2+ paradox injury mediated through TRPC channels in mouse ventricular myocytes. *Br. J. Pharmacol.* 161 1734–1750. 10.1111/j.1476-5381.2010.00986.x 20718730 PMC3010579

[B18] Kojima A. Kitagawa H. Omatsu-Kanbe M. Matsuura H. Nosaka S. (2012). Presence of store-operated Ca2+ entry in C57BL/6J mouse ventricular myocytes and its suppression by sevoflurane. *Br. J. Anaesth.* 109 352–360. 10.1093/bja/aes212 22777657

[B19] Kurebayashi N. Ogawa Y. (2001). Depletion of Ca2+ in the sarcoplasmic reticulum stimulates Ca2+ entry into mouse skeletal muscle fibres. *J. Physiol.* 533(Pt 1) 185–199. 10.1111/j.1469-7793.2001.0185b.x 11351027 PMC2278591

[B20] Kuwahara K. Wang Y. McAnally J. Richardson J. A. Bassel-Duby R. Hill J. A. (2006). TRPC6 fulfills a calcineurin signaling circuit during pathologic cardiac remodeling. *J. Clin. Invest.* 116 3114–3126. 10.1172/JCI27702 17099778 PMC1635163

[B21] Leuner K. Kazanski V. Muller M. Essin K. Henke B. Gollasch M. (2007). Hyperforin–a key constituent of St. John’s wort specifically activates TRPC6 channels. *FASEB J.* 21 4101–4111. 10.1096/fj.07-8110com 17666455

[B22] Liao Y. Erxleben C. Abramowitz J. Flockerzi V. Zhu M. X. Armstrong D. L. (2008). Functional interactions among Orai1, TRPCs, and STIM1 suggest a STIM-regulated heteromeric Orai/TRPC model for SOCE/Icrac channels. *Proc. Natl. Acad. Sci. U.S.A.* 105 2895–2900. 10.1073/pnas.0712288105 18287061 PMC2268556

[B23] Liao Y. Plummer N. W. George M. D. Abramowitz J. Zhu M. X. Birnbaumer L. (2009). A role for orai in TRPC-mediated Ca2+ entry suggests that a TRPC:Orai complex may mediate store and receptor operated Ca2+ entry. *Proc. Natl. Acad. Sci. U.S.A.* 106 3202–3206. 10.1073/pnas.0813346106 19221033 PMC2651283

[B24] Makarewich C. A. Zhang H. Davis J. Correll R. N. Trappanese D. M. Hoffman N. E. (2014). Transient receptor potential channels contribute to pathological structural and functional remodeling after myocardial infarction. *Circ. Res.* 115 567–580. 10.1161/CIRCRESAHA.115.303831 25047165 PMC4149870

[B25] Mohl M. C. Iismaa S. E. Xiao X. H. Friedrich O. Wagner S. Nikolova-Krstevski V. (2011). Regulation of murine cardiac contractility by activation of alpha(1A)-adrenergic receptor-operated Ca(2+) entry. *Cardiovasc. Res.* 91 310–319. 10.1093/cvr/cvr081 21546445

[B26] Ong H. L. Cheng K. T. Liu X. Bandyopadhyay B. C. Paria B. C. Soboloff J. (2007). Dynamic assembly of TRPC1-STIM1-Orai1 ternary complex is involved in store-operated calcium influx. evidence for similarities in store-operated and calcium release-activated calcium channel components. *J. Biol. Chem.* 282 9105–9116. 10.1074/jbc.M608942200 17224452 PMC3309402

[B27] Parekh A. B. Putney J. W. Jr. (2005). Store-operated calcium channels. *Physiol. Rev.* 85 757–810. 10.1152/physrev.00057.2003 15788710

[B28] Putney J. W. Jr. (1986). A model for receptor-regulated calcium entry. *Cell Calcium* 7 1–12. 10.1016/0143-4160(86)90026-6 2420465

[B29] Rohrig T. Pacjuk O. Hernandez-Huguet S. Korner J. Scherer K. Richling E. (2017). Inhibition of cyclic adenosine monophosphate-specific phosphodiesterase by various food plant-derived phytotherapeutic agents. *Medicines* 4:E80. 10.3390/medicines4040080 29113064 PMC5750604

[B30] Ross G. Bajwa T. J. Edwards S. Emelyanova L. Rizvi F. Holmuhamedov E. (2017). Enhanced store-operated Ca2+ influx and ORAI1 expression in ventricular fibroblasts from human failing heart. *Biol. Open.* 6 326–332. 10.1242/bio.022632 28126709 PMC5374400

[B31] Sabourin J. Bartoli F. Antigny F. Gomez A. M. Benitah J. P. (2016). Transient receptor potential canonical (TRPC)/orai1-dependent store-operated Ca2+ channels: new targets of aldosterone in cardiomyocytes. *J. Biol. Chem.* 291 13394–13409. 10.1074/jbc.M115.693911 27129253 PMC4933247

[B32] Sabourin J. Robin E. Raddatz E. (2011). A key role of TRPC channels in the regulation of electromechanical activity of the developing heart. *Cardiovasc. Res.* 92 226–236. 10.1093/cvr/cvr167 21672930

[B33] Selvaraj S. Sun Y. Singh B. B. (2010). TRPC channels and their implication in neurological diseases. *CNS Neurol. Disord. Drug Targets* 9 94–104. 10.2174/187152710790966650 20201820 PMC2846610

[B34] Shin D. W. Pan Z. Kim E. K. Lee J. M. Bhat M. B. Parness J. (2003). A retrograde signal from calsequestrin for the regulation of store-operated Ca2+ entry in skeletal muscle. *J. Biol. Chem.* 278 3286–3292. 10.1074/jbc.M209045200 12419813

[B35] Trepakova E. S. Gericke M. Hirakawa Y. Weisbrod R. M. Cohen R. A. Bolotina V. M. (2001). Properties of a native cation channel activated by Ca2+ store depletion in vascular smooth muscle cells. *J. Biol. Chem.* 276 7782–7790. 10.1074/jbc.M010104200 11113149

[B36] Tu P. Gibon J. Bouron A. (2010). The TRPC6 channel activator hyperforin induces the release of zinc and calcium from mitochondria. *J. Neurochem.* 112 204–213. 10.1111/j.1471-4159.2009.06446.x 19845832

[B37] Uemura A. Naito Y. Matsubara T. (2002). Dynamics of Ca(2+)/calmodulin-dependent protein kinase II following acute myocardial ischemia-translocation and autophosphorylation. *Biochem. Biophys. Res. Commun.* 297 997–1002. 10.1016/S0006-291X(02)02279-9 12359253

[B38] Vazquez G. Lievremont J. P. Bird G. J. Putney J. W. Jr. (2001). Human Trp3 forms both inositol trisphosphate receptor-dependent and receptor-independent store-operated cation channels in DT40 avian B lymphocytes. *Proc. Natl. Acad. Sci. U.S.A.* 98 11777–11782. 10.1073/pnas.201238198 11553786 PMC58806

[B39] Vazquez G. Wedel B. J. Trebak M. St John Bird G. Putney J. W. Jr. (2003). Expression level of the canonical transient receptor potential 3 (TRPC3) channel determines its mechanism of activation. *J. Biol. Chem.* 278 21649–21654. 10.1074/jbc.M302162200 12686562

[B40] Venkatachalam K. Ma H. T. Ford D. L. Gill D. L. (2001). Expression of functional receptor-coupled TRPC3 channels in DT40 triple receptor InsP3 knockout cells. *J. Biol. Chem.* 276 33980–33985. 10.1074/jbc.C100321200 11466302

[B41] Venkatachalam K. Montell C. (2007). TRP channels. *Annu. Rev. Biochem.* 76 387–417. 10.1146/annurev.biochem.75.103004.142819 17579562 PMC4196875

[B42] Voelkers M. Salz M. Herzog N. Frank D. Dolatabadi N. Frey N. (2010). Orai1 and Stim1 regulate normal and hypertrophic growth in cardiomyocytes. *J. Mol. Cell Cardiol.* 48 1329–1334. 10.1016/j.yjmcc.2010.01.020 20138887 PMC5511312

[B43] Wit A. L. Janse M. J. (1992). Experimental models of ventricular tachycardia and fibrillation caused by ischemia and infarction. *Circulation* 1(Suppl) I32–I42. 1728503

[B44] Wu X. Eder P. Chang B. Molkentin J. D. (2010). TRPC channels are necessary mediators of pathologic cardiac hypertrophy. *Proc. Natl. Acad. Sci. U.S.A.* 107 7000–7005. 10.1073/pnas.1001825107 20351294 PMC2872458

[B45] Xie L. H. Weiss J. N. (2009). Arrhythmogenic consequences of intracellular calcium waves. *Am. J. Physiol. Heart Circ. Physiol.* 297 H997–H1002. 10.1152/ajpheart.00390.2009 19561309 PMC2755983

[B46] Zhao Z. Gordan R. Wen H. Fefelova N. Zang W. J. Xie L. H. (2013). Modulation of intracellular calcium waves and triggered activities by mitochondrial ca flux in mouse cardiomyocytes. *PLoS One* 8:e80574. 10.1371/journal.pone.0080574 24348912 PMC3857829

[B47] Zhao Z. Kudej R. K. Wen H. Fefelova N. Yan L. Vatner D. E. (2018). Antioxidant defense and protection against cardiac arrhythmias: lessons from a mammalian hibernator (the woodchuck). *FASEB J.* 32 4229–4240. 10.1096/fj.201701516R 29490168 PMC6044062

[B48] Zhao Z. Wen H. Fefelova N. Allen C. Baba A. Matsuda T. (2012). Revisiting the ionic mechanisms of early afterdepolarizations in cardiomyocytes: predominant by Ca waves or Ca currents? *Am. J. Physiol. Heart Circ. Physiol.* 302 H1636–H1644. 10.1152/ajpheart.00742.2011 22307670 PMC3330805

